# A rapid multiplex platform for simultaneous detection of chikungunya virus, dengue virus, and dengue serotyping based on isothermal amplification and lateral flow dipsticks

**DOI:** 10.1186/s40249-026-01450-9

**Published:** 2026-05-09

**Authors:** Gaowen Liu, Xinlin Wu, Yingchao Chang, Mengyuan Zheng, Li Liu, Xueshan Xia, Yue Feng

**Affiliations:** 1 Faculty of Life Science and Technology & Yunnan Provincial Key Laboratory of Public Health and Biosafety, Kunming University of Science and Technology, No.727 Jingming Road, Chenggong Campus, Kunming, Yunnan China; 2 Yunnan Kecan Biotechnology Co., Ltd, Kunming, China; 3 Yunnan Provincial Key Laboratory of Public Health and Biosafety, Kunming Medical University, Kunming, Yunnan China

**Keywords:** Dengue virus, Chikungunya virus, Multienzyme isothermal rapid amplification, Lateral flow dipstick strips, Serotype genotyping

## Abstract

**Background:**

The expeditious and precise diagnosis of dengue virus (DENV) and chikungunya virus (CHIKV) is paramount for effective patient management and the control of outbreaks. In this study, a duplex reverse transcription multi-enzyme isothermal amplification (RT-MIRA) assay was established for the simultaneous detection of DENV and CHIKV, followed by a nested RT-MIRA assay for DENV serotyping (DENV-1 to -4).

**Methods:**

Specific primers and probes targeting the DENV 3′-UTR, CHIKV E1 gene, and four DENV serotypes were designed. The duplex RT-MIRA and nested DENV RT-MIRA serotyping reaction systems were optimized at 39 °C with portable fluorescence or lateral flow dipstick readouts. For methodological validation, specificity was evaluated against 35 related pathogens, and the 95% limit of detection (LOD95) was determined via probit regression. For clinical validation, serum samples from 236 suspected patients were tested, benchmarking against RT-qPCR and serology. Statistical analyses included the Wilson score method for calculating 95% confidence intervals (*CIs*) and Cohen’s kappa (*κ*). For external verification, 12 CHIKV-positive clinical samples and 5 artificially simulated co-infection samples were retrospectively analyzed to validate assay accuracy.

**Results:**

The duplex RT-MIRA assay exhibited no cross-reactivity with other pathogens. The LOD95 values were 13.47 copies/μl for DENV and 10.49 copies/μl for CHIKV. Clinical validation demonstrated sensitivities of 96.15% (95% *CI*: 89.28%–98.67%) for DENV and 88.89% (95% *CI*: 67.20%–96.90%) for CHIKV. Specificity was 100% (95% *CI*: 92.87%–100%) for both. Agreement with RT-qPCR was strong for DENV (*κ* = 0.96) and CHIKV (*κ* = 0.92). The nested RT-MIRA serotyping assay showed high sensitivity (LOD95: 1.6–18.7 copies/μl) without cross-reactivity, accurately differentiating 75 DENV-positive samples into 71 DENV-1 and 4 DENV-2. In the external verification, the assay accurately detected 10 CHIKV mono-infections and 2 CHIKV/DENV co-infections, and distinguished four DENV serotypes in simulated matrices.

**Conclusions:**

A rapid and sensitive integrated method has been developed that combines duplex RT-MIRA for detecting DENV and CHIKV, and nested RT-MIRA for serotyping DENV. The simplicity and speed of the amplification and detection steps demonstrate this platform's potential for use in point-of-care testing and surveillance in areas with limited resources, particularly when used alongside portable extraction methods.

**Supplementary Information:**

The online version contains supplementary material available at 10.1186/s40249-026-01450-9.

## Background

Dengue virus (DENV) and chikungunya virus (CHIKV) are arthropod-borne viruses transmitted primarily by *Aedes aegypti* and *Aedes albopictus* mosquitoes in tropical and subtropical regions, constituting a global public health threat. The clinical presentation of dengue fever is characterized by a sudden onset of high fever, severe headache, intense muscle pain, joint pain, a maculopapular rash, and leukopenia. In contrast, chikungunya infection is characterized by a sudden onset of high fever and debilitating joint pain that typically affects small joints symmetrically, along with muscle pain, a maculopapular rash, headache, and conjunctivitis. As of April 2024, DENV cases exceeded 7.6 million worldwide, with 16,000 severe cases and 3000 deaths [1]. By September 2025, CHIKV had spread to 119 countries, causing 181,679 confirmed cases and 155 deaths, mainly in the Americas [2, 3]. The manifestation of symptoms in both viruses is analogous, which can result in erroneous diagnoses [4, 5]. DENV is comprised of four serotypes, and the phenomenon of antibody-dependent enhancement contributes to severe cases [6]. Presently, there are no specific antiviral treatments available, and the number of vaccine options is limited. This scenario underscores the imperative for advanced diagnostic methodologies to address the complexity of such infections.

Laboratory diagnostics for DENV and CHIKV primarily rely on serological assays and nucleic acid amplification tests (NAATs). Serological tests, including Enzyme-linked immunosorbent assay (ELISA) and rapid tests, are utilized for the detection of Immunoglobulin M (IgM) and Immunoglobulin G (IgG) [7]. These antibodies fulfill a pivotal function in the diagnostic process during the convalescent phase, aiding in the differentiation between primary and secondary infections. However, cross-reactivity among flaviviruses has the potential to result in false positives, thereby complicating the process of DENV serotype identification [8, 9]. The DENV non-structural protein 1(NS1) antigen assay is a valuable tool for acute-phase screening. However, its sensitivity can be diminished in secondary infections due to the presence of immune complexes [10]. A substantial body of research has demonstrated that NAATs, particularly reverse transcription-quantitative polymerase chain reaction (RT-qPCR), are regarded as the gold standard for early diagnosis due to their high sensitivity and specificity [11]. Multiplex assays have been developed to detect all DENV serotypes and CHIKV simultaneously [12]. Isothermal amplification technologies, including reverse transcription loop-mediated isothermal amplification (RT-LAMP), recombinase polymerase amplification (RPA), and reverse transcription multienzyme isothermal rapid amplification (RT-MIRA), clustered regularly interspaced short palindromic repeats (CRISPR)-Cas-based system, offer fast results for point-of-care testing (POCT) [13, 14]. However, these technologies are encumbered by substantial costs and complexity. The development of novel detection methodologies is imperative for effectively differentiating between DENV, CHIKV, and their serotypes.

In this study, we established a duplex RT-MIRA assay for rapid and ultrasensitive discrimination of DENV and CHIKV infections, along with simultaneous DENV serotyping. This method is characterized by its reliability and cost-effectiveness, rendering it optimal for on-site identification of DENV and CHIKV infections, as well as for disease surveillance and control.

## Materials and methods

### Preparation of RNA standards

Standard RNA transcripts for pan-DENV, DENV1-4, and CHIKV were synthesized via in vitro transcription. The T7 High Yield RNA Transcription Kit (Vazyme, Nanjing, China) was utilized to transcribe RNA, which was subsequently quantified using the Qubit version 4.0 (Thermo Fisher Scientific, USA). The RNA standards were serially diluted ten-fold and served as templates for subsequent experiments.

### Cells and viruses

Baby hamster kidney (BHK-21) cells were cultured in Dulbecco’s Modified Eagle Medium at 37 °C. In contrast, *Aedes albopictus* mosquito C6/36 cells were grown in RPMI-1640 medium at 28 °C. Both cell lines were cultured in media supplemented with 10% fetal bovine serum and 100 U/ml penicillin–streptomycin under a 5% CO2 atmosphere. Four strains of the DENV were propagated using the C6/36 cell line: DENV-1, DENV-2, DENV-3, and DENV-4. In addition, three genotypes of CHIKV, encompassing ECSA, Asian, and West African strains, were propagated in BHK-21 cells. The virus titers were subsequently assessed using a plaque assay in BHK-21 cells. The thirty-five pathogens utilized for the specificity evaluation were obtained from the Yunnan Provincial Key Laboratory of Public Health and Biosafety (Table S1).

### Design of the duplex RT-MIRA

CHIKV-specific MIRA primers and probes were designed with modifications as previously described [15]. The pan-DENV primers and probes targeted the 3′ untranslated region (3’-UTR), while the serotype-specific primers and probes targeted the 5′ untranslated region(5’-UTR), pre-membrane protein, and capsid protein gene. The design of the primers and probes was accomplished with the utilization of Primer Premier 5.0 (Premier Biosoft International, San Francisco, CA, USA), with their subsequent synthesis being carried out by Taihe Biotechnology Co., Ltd (Beijing, China). The sequences are listed in Tables S2 and S3.

### Duplex RT-MIRA assay for DENV and CHIKV

Nucleic acids were extracted from reference pathogens and archived clinical samples using the GeneRotex system and Ex-DNA/RNA (4.0) kit (Tianlong, Xi'an, China), following the manufacturer's instructions. The duplex RT-MIRA assay was performed using primer and probe sets optimized for DENV and CHIKV. Briefly, the 50 μl reaction mixture consisted of 29.4 μl of buffer A, 2.0 μl of each primer (10 μmol/L), 0.6 µl of each probe (10 μmol/L), 2.5 µl of buffer B, 3.9 µl of nuclease-free water, and 5 µl of RNA template. Amplification was carried out at 39 °C for 20 min, and results were read using a portable fluorescence reader or LFD strips. DENV serotyping employed a nested MIRA strategy, comprising an outer RT-MIRA and an inner MIRA step, as previously described [15]. Reagents for the inner MIRA amplification were immobilized in the tube cap, spatially separated from the outer RT-MIRA reaction mixture at the bottom. The first round of amplification was performed at 39 °C for 10 min, followed by centrifugation to mix the inner reagents with the primary amplification products. This initiated the second round of amplification at 39 °C for 20 min, allowing the entire amplification process to be completed without opening the tube. Results were read using a portable fluorescence reader or LFD strips.

### Evaluation of specificity, sensitivity, reproducibility, and accuracy

A total of 35 pathogens, including 22 viruses, 7 bacteria, 3 yeasts, and 3 fungi, were tested (Table S1). The limit of detection (LOD) was determined using ten-fold serial dilutions of pan-DENV, DENV 1–4, and CHIKV RNA standards (from 10^4^ to 1 copy/µl) in three independent replicates. Intra-assay and inter-assay repeatability were measured using a 10^3^ copies/µl RNA standard, with assays performed on alternate days. Finally, laboratory-stored positive and negative samples, along with artificially synthesized mixed-infection samples, were tested.

### Evaluation of stability and interference

Lyophilized reagents were stored at 4 °C, 25 °C, and 37 °C for up to 14 days, and subsequently tested using negative serum spiked with inactivated DENV and CHIKV cultures. Additionally, inactivated viral cultures were tested in the presence of varying concentrations of endogenous substances (hemoglobin and albumin) and exogenous substances, including anticoagulants (heparin, EDTA, and sodium citrate) and common therapeutic drugs (ribavirin, acetaminophen, amoxicillin, and dexamethasone).

### Clinical samples

A total of 236 clinical serum samples from suspected patients were collected from imported cases at Kunming Changshui International Airport by Yunnan International Travel Health Care Center between 2019 and 2020. The study population included travelers presenting with acute fever (> 37.5 °C) or clinical manifestations suggestive of arboviral infection (e.g., rash, arthralgia, or headache). Samples with insufficient volume (< 200 µl) or lacking essential clinical data regarding symptom onset were excluded. All the samples were stored at − 80 °C for RNA extraction.

All samples were subjected to a rigorous diagnostic algorithm, including RT-qPCR, IgM capture ELISA, and NS1 antigen detection, which served as the reference standard. Based on these reference results, the cohort included confirmed positive cases for DENV and CHIKV, as well as healthy controls and other febrile illness cases confirmed to be negative. The demographic and clinical characteristics of the study cohort were analyzed to assess representativeness. As detailed in Fig. S1, the patients covered a broad age range with a balanced gender distribution. The most common clinical symptoms observed were fever and rash, consistent with typical arboviral disease presentations. This well-characterized panel was subsequently used to evaluate the diagnostic performance (sensitivity, specificity, and concordance) of the duplex RT-MIRA assay.

### External validation

External validation was independently conducted by the Public Health and Biosafety Laboratory of Yunnan Province. A retrospective analysis was performed using 12 clinical samples identified at Kunming Changshui International Airport between 2019 and 2020. These samples comprised 10 cases of CHIKV mono-infection and 2 cases of CHIKV/DENV co-infection. Additionally, artificially simulated co-infection samples were prepared by mixing five human serum samples, previously confirmed positive for the four DENV serotypes, with CHIKV mono-positive plasma. All validation procedures were executed exclusively by the staff of the aforementioned laboratory.

### Statistical analysis

Statistical analyses and figure generation were performed using GraphPad Prism 8.0.1 (GraphPad Software, San Diego, CA, USA), OriginPro 2025 (OriginLab Corporation, Northampton, MA, USA), and R 4.5.2 (R Foundation for Statistical Computing, Vienna, Austria). Experiments were conducted in triplicate, with data presented as mean ± standard deviation (*SD*). The 95% limit of detection (LOD95) was estimated via probit regression. For clinical validation, diagnostic sensitivity (true positives/[true positives + false negatives]) and specificity (true negatives/[true negatives + false positives]) were calculated, with 95% confidence intervals (*CIs*) determined using the Wilson score method. Agreement between the duplex RT-MIRA assay and reference methods was assessed using Cohen’s kappa coefficient (*κ*). To evaluate the statistical significance of differences in paired binary outcomes, McNemar’s test was employed. A *P-value* < 0.05 was considered statistically significant.

## Results

### Overall workflow from sample to signal

**Fig. 1:**
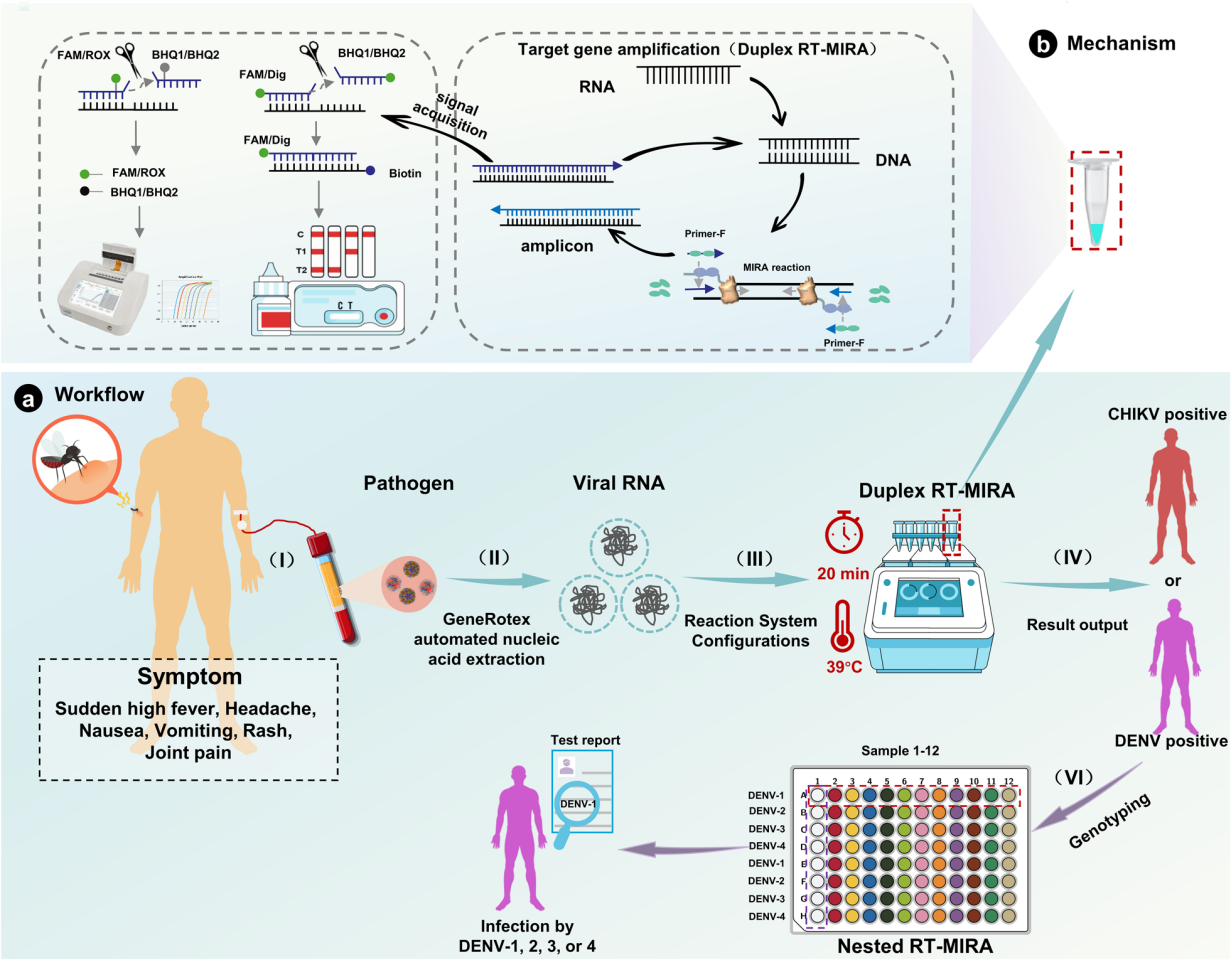
Schematic illustration of the duplex RT-MIRA workflow and operational principle. **a** Overview of the workflow from sample to signal. Serum samples are collected from suspected DENV and CHIKV patients (I); nucleic acid extraction is performed (II); extracted RNA is added to the duplex RT-MIRA reaction system and incubated at 39 °C for 20 min (III); results are interpreted (IV). For DENV-positive samples, nested RT-MIRA is further conducted for serotyping to determine the infecting DENV serotype (IV). **b** Schematic diagram of the duplex RT-MIRA principle. Viral RNA is first reverse-transcribed into complementary DNA (cDNA). Recombinase facilitates the binding of specific primers to the cDNA template, forming nucleoprotein complexes. Under the synergistic action of helicase, single-stranded DNA-binding proteins, and strand-displacing DNA polymerase, the target gene is exponentially amplified. Meanwhile, a fluorophore-labeled probe hybridizes with the template, and exonuclease III (exo) or exonuclease IV (nfo) specifically recognizes the THF site on the probe, thereby releasing a fluorescent signal or forming a fluorescently labeled amplicon. Amplification results can be interpreted in real time using a portable fluorescence reader or detected at the endpoint using a lateral flow device (LFD). RT-MIRA: Reverse transcription multienzyme isothermal amplification; CHIKV: Chikungunya virus; DENV: Dengue virus

The workflow for the duplex RT-MIRA assay targeting CHIKV and DENV is illustrated in Fig. 1. Serum samples from suspected patients are collected, and viral RNA is extracted as the template for amplification. The duplex RT-MIRA amplification process is carried out at a constant temperature of 39 °C for a duration of 20 min, during which the viral RNA is reverse-transcribed into cDNA. Recombinase enzymes facilitate the binding of specific primers to the cDNA, thereby forming nucleoprotein complexes. The process of exponentially amplifying target genes involves the use of helicase, single-stranded DNA-binding proteins, and strand-displacing DNA polymerase. Fluorophore-labeled probes hybridize to the template, and exonuclease enzymes subsequently release a fluorescent signal. The results of these tests can be interpreted in real-time using a portable fluorescence reader or as endpoint measurements with LFD. DENV-positive samples undergo a nested RT-MIRA assay for serotyping to identify the infecting serotype.

### Establish a duplex RT-MIRA assay

**Fig. 2:**
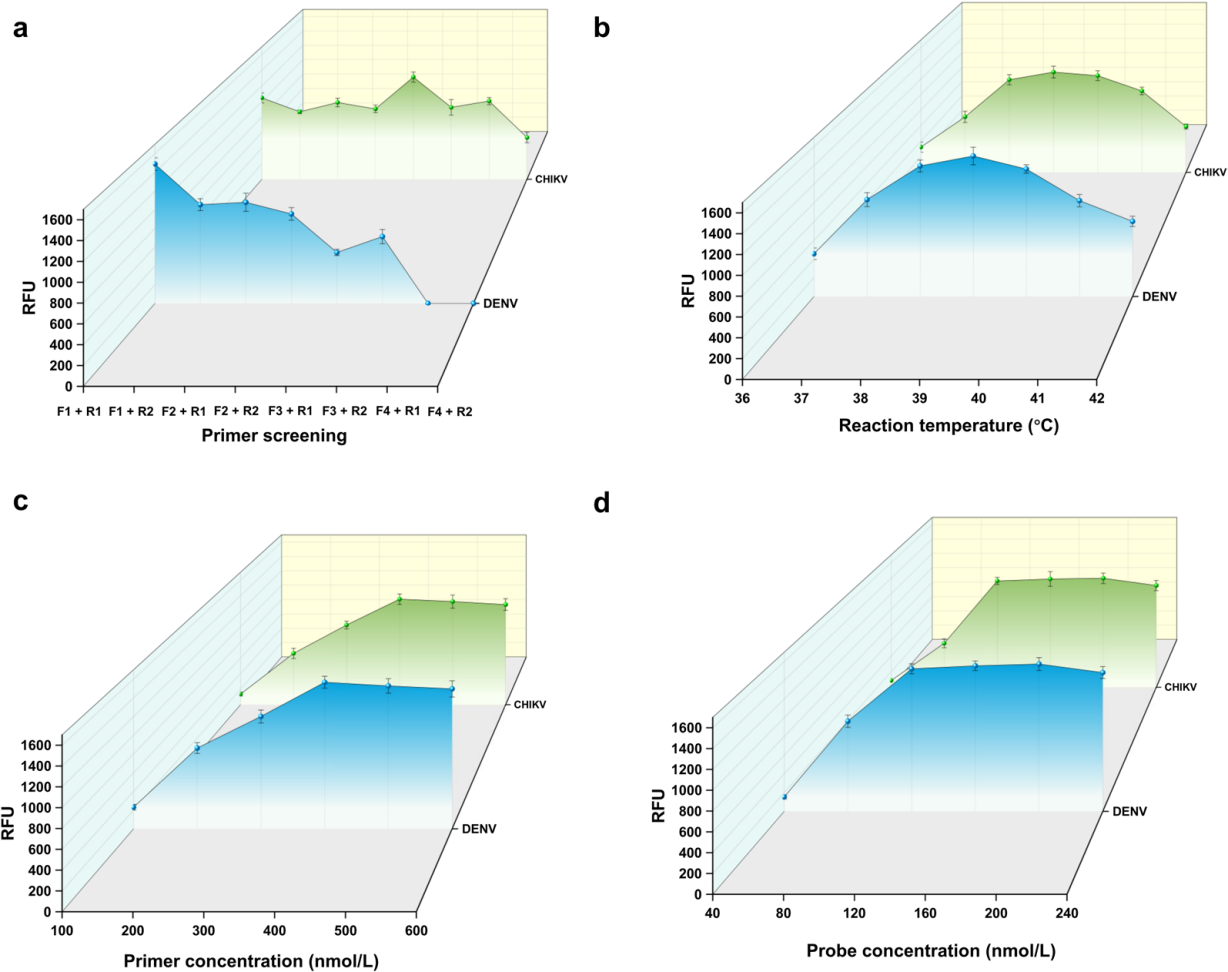
Optimization of reaction conditions for the duplex RT-MIRA detection system. **a** Multiplex MIRA primers for DENV and CHIKV were designed, and primer screening was performed using RNA standards at a concentration of 1 × 10^4^ copies/μl as the template. **b**–**d** Using RNA standards at the same concentration (1 × 10^4^ copies/μl), key factors affecting duplex RT-MIRA amplification efficiency—including temperature, primer concentration, and probe concentration—were systematically optimized. RT-MIRA: Reverse transcription multienzyme isothermal amplification; CHIKV: Chikungunya virus; DENV: Dengue virus

The preliminary phase in the construction of a duplex RT-MIRA detection system entailed the screening of optimal primer combinations and the optimization of reaction conditions. Primers and probes targeting the DENV 3′UTR and CHIKV E1 gene were designed and tested with RNA standards at a concentration of 1 × 10^4^ copies/μl. The MIRA primer sets F1 + R1 for DENV and F3 + R1 for CHIKV demonstrated the highest amplification efficiency (Fig. 2a). Optimal conditions were determined to be 39 °C, 400 nmol/L primer, and 120 nmol/L probe concentrations, yielding maximal efficiency and the strongest fluorescence signals (Fig. 2b–d). Consequently, a duplex RT-MIRA assay was developed for the concurrent detection of DENV and CHIKV.

### Evaluation of the specificity and sensitivity of the duplex RT-MIRA assay

**Fig. 3:**
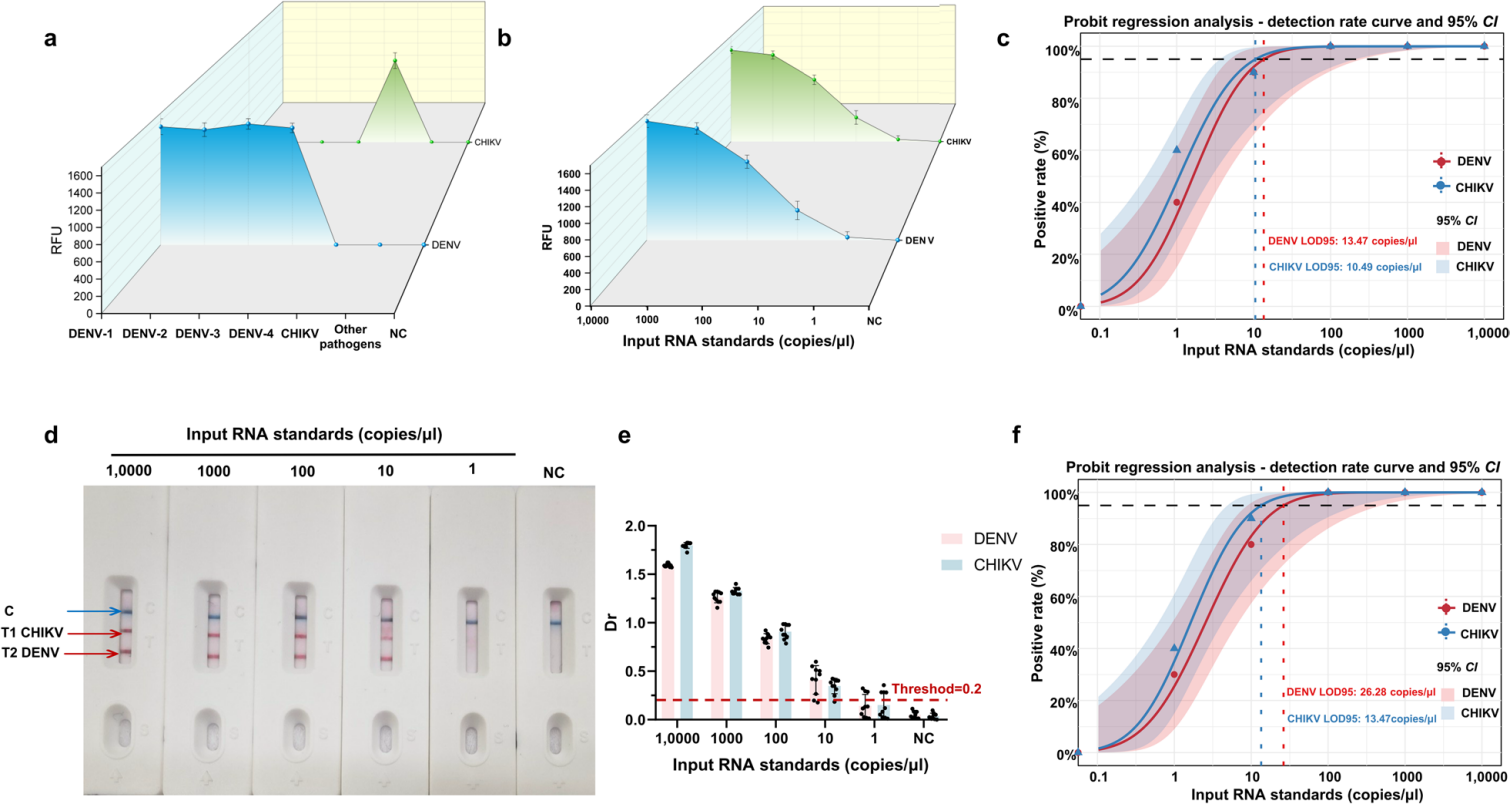
Specificity and sensitivity evaluation of the duplex RT-MIRA detection system. **a** Specificity assessment of duplex RT-MIRA detection using 35 pathogen species (partial results shown). **b**, **d**, **e** Analytical sensitivity of the duplex RT-MIRA system for detecting dengue virus (DENV) and chikungunya virus (CHIKV) was evaluated using RNA standard samples at concentrations ranging from 1 × 10^4^ copies/μl to 1 copy/μl, with both fluorescence detection and LFD methods employed. **c**, **f** Probability regression plots comparing fluorescence detection and LFD: The red curve and pink shaded region represent DENV and its 95% *Cl*, while the blue curve and light blue region represent CHIKV and its 95% *CI*. The observed intersection points of the black dashed line with the red or blue dashed lines indicate the estimated detection limits at 95% detection (LOD95) for each method. Each concentration was tested in10 parallel replicates. RT-MIRA: Reverse transcription multienzyme isothermal amplification

The evaluation of specificity was conducted through the testing of 35 pathogens associated with febrile illnesses that are analogous to those induced by DENV and CHIKV, to determine their cross-reactivity. The findings demonstrated a complete absence of cross-reactivity, thereby substantiating the remarkable specificity of the duplex RT-MIRA assay (Fig. 3a, Table S1). To assess analytical sensitivity, tenfold serial dilutions of standard RNA (1 × 10^4^ copies/μl to 1 copy/μl) were analyzed in 10 replicates. A 100% detection rate was observed at concentrations ≥ 100 copies/μl, with a decline in detection as RNA concentrations decreased (Fig. 3b–e). The LOD95 was determined to be 13.47 copies/μl for DENV and 10.49 copies/μl for CHIKV by fluorescence detection (Fig. 3c, f). These results confirm the duplex RT-MIRA assay's high sensitivity for simultaneous detection of DENV and CHIKV.

### Performance evaluation of a nested RT-MIRA assay

For DENV serotyping, a nested RT-MIRA assay was employed, as previously described in a separate study. This assay utilized optimal specific primer sets targeting all four DENV serotypes (Fig. S2). The amplification process was conducted at a temperature of 39 °C, with the initial 10-min phase occurring in an outer RT-MIRA reaction and a subsequent 20-min phase within the inner MIRA reaction.

**Fig. 4:**
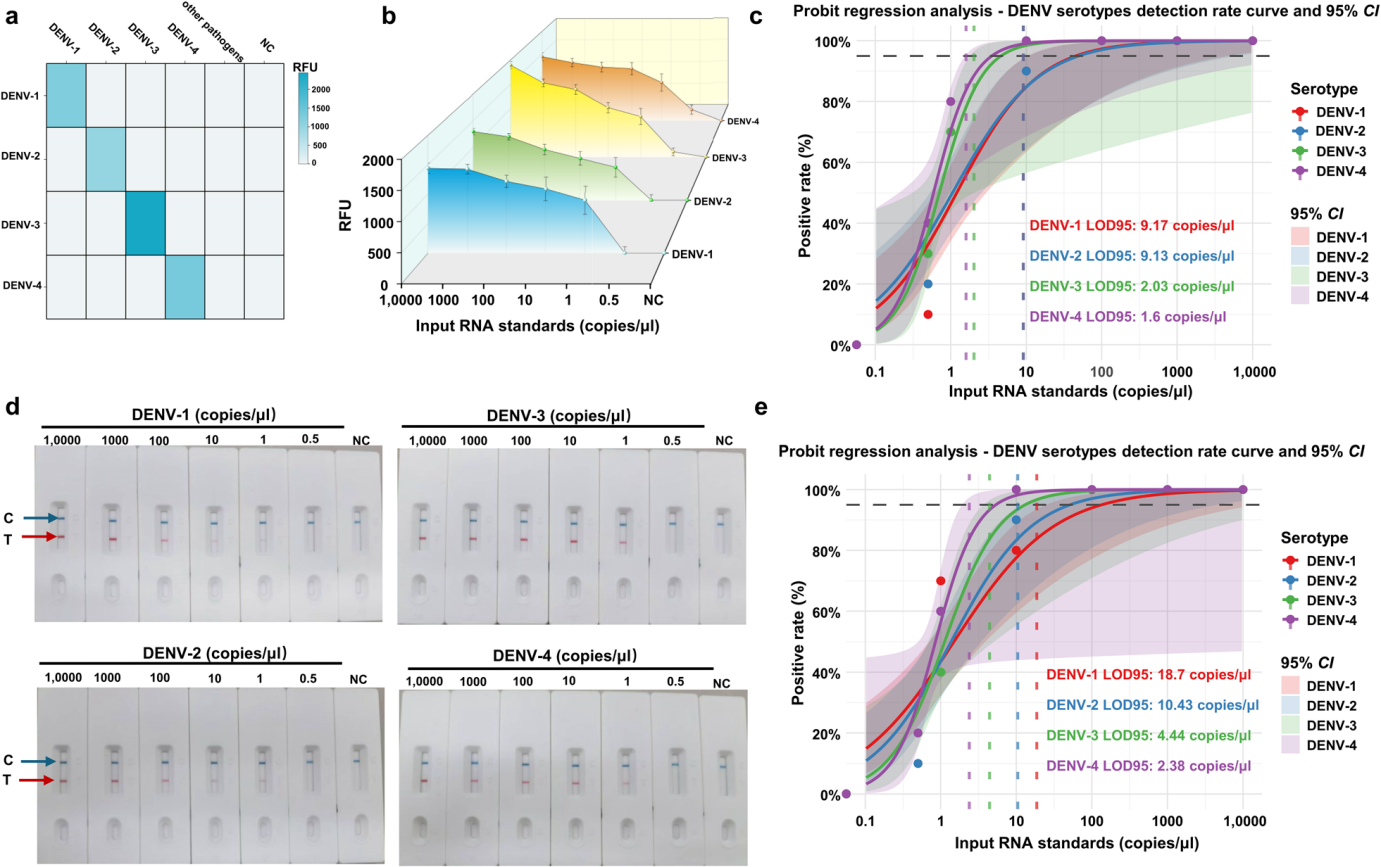
Specificity and sensitivity evaluation of nested RT-MIRA for dengue virus serotyping. **a** Specificity assessment of nested RT-MIRA detection using 35 pathogen species (partial results shown). **b**, **d** Analytical sensitivity of nested RT-MIRA for the four dengue virus (DENV) serotypes was evaluated using RNA standard samples at concentrations ranging from 1 × 10^4^ copies/μl to 1 copy/μl, with both fluorescence detection and LFD methods applied. **c**, **e** Probability regression plots for fluorescence detection and LFD: curves and shaded regions in different colors represent the four DENV serotypes and their respective 95% *CIs*. The intersection points of the black dashed line with the colored dashed lines indicate the estimated 95% limit of detection (LOD95) for each DENV serotype. Each concentration was tested in 10 parallel replicates. RT-MIRA: Reverse transcription multienzyme isothermal amplification; *CI*: Confidence interval

The assay's analytical performance was evaluated for specificity and sensitivity. The results demonstrated no cross-reactivity with 34 other tested pathogens, thereby confirming the specificity of each serotype (Fig. 4a, Table S1). Sensitivity was gauged through the implementation of tenfold serial dilutions of RNA, with a detection limit as low as 1 copy/μl observed for all serotypes (Fig. 4b, d). Subsequent tests established the LOD95, which differed for each serotype, ranging from 1.6 to 18.7 copies/μl, contingent on the utilized detection methodologies (Fig. 4c, e). These findings confirm the high sensitivity and reliability of the nested RT-MIRA assay for dengue virus serotyping.

### Evaluation of repeatability and accuracy

The repeatability and accuracy of the duplex RT-MIRA and nested RT-MIRA assays were rigorously evaluated. As shown in Fig. S3, the intra-assay coefficient of variation (*CV*) ranged from 1.99% to 3.19%, while the inter-assay *CV* ranged from 3.68% to 4.25%, all remaining well below the 5% threshold. Clinical performance was validated using 138 samples, and the results showed 100% concordance with the reference qPCR method (Table 1). Specifically, the duplex RT-MIRA correctly identified 98 DENV-positive and 28 CHIKV-positive cases. Furthermore, the nested RT-MIRA achieved 100% accuracy in serotyping compared to qPCR, successfully identifying all 10 cases of mixed DENV serotype infections and 8 cases of DENV/CHIKV co-infection. Notably, for the DENV-2 serotype, both the nested RT-MIRA and qPCR consistently identified 19 positive cases out of 20 samples, further confirming the high reliability of the developed MIRA platform.

**Table 1:** Accuracy evaluation of the duplex RT-MIRA for detecting DENV and CHIKV, as well as serotyping of DENV using the nested RT-MIRA assay

Sample Type	Sample quantity	Duplex RT-MIRA detection		Nested RT-MIRA serotyping				RT-PCR serotyping			
		DENV Positive	CHIKV Positive	DENV-1 Positive	DENV-2 Positive	DENV-3 Positive	DENV-4 Positive	DENV-1 Positive	DENV-2 Positive	DENV-3 Positive	DENV-4 Positive
DENV-1	20	20	–	20	–	–	–	20	–	–	–
DENV-2	20	20	–	–	19	–	–	–	19	–	–
DENV-3	20	20	–	–	–	20	–	–	–	20	–
DENV-4	20	20	–	–	–	–	20	–	–	–	20
DENV-1 + DENV-2	2	2	–	2	2	–	–	2	2	–	–
DENV-1 + DENV 3	2	2	–	2	–	2	–	2	–	2	–
DENV-1 + DENV-4	2	2	–	2	–	–	2	2	–	–	2
DENV-2 + DENV-3	2	2	–	–	2	2	–	–	2	2	–
DENV-2 + DENV-4	2	2	–	–	2	–	2	–	2	–	2
CHIKV	20	–	20	–	–	–	–	–	–	–	–
DENV-1 + CHIKV	2	2	2	2	–	–	–	2	–	–	–
DENV-2 + CHIKV	2	2	2	–	2	–	–	–	2	–	–
DENV-3 + CHIKV	2	2	2	–	–	2	–	–	–	2	–
DENV-4 + CHIKV	2	2	2	–	–	–	2	–	–	–	2
Negative Sample	20	–	–	–	–	–	–	–	–	–	–

RT-MIRA: Reverse transcription multienzyme isothermal amplification; CHIKV: Chikungunya virus; DENV: Dengue virus

### Stability and interference analysis

The stability of the lyophilized reagents was evaluated using simulated clinical samples. Results indicated that after storage at 4 °C, 25 °C, and 37 °C for up to 14 days, the assay consistently maintained its detection capability for both DENV and CHIKV, demonstrating excellent reagent stability (Table S4). In addition, the assay demonstrated strong robustness against common matrix interferences. No significant inhibition or cross-reactivity was observed in samples containing high concentrations of hemoglobin, albumin, or anticoagulants (heparin, EDTA, and sodium citrate), nor in the presence of common therapeutic drugs (Table S5).

### Validation by clinical sample

**Fig. 5:**
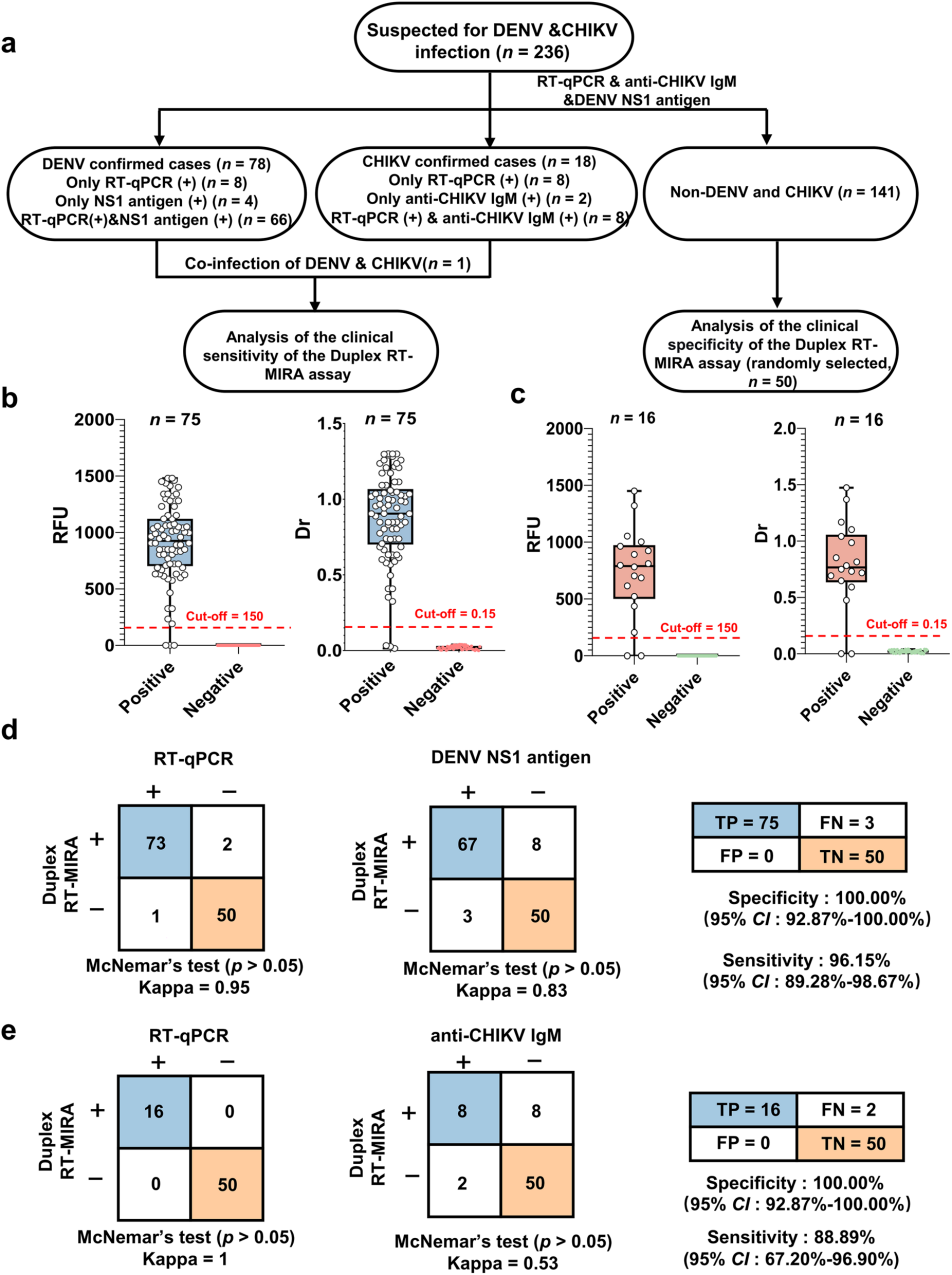
Clinical evaluation of duplex RT-MIRA for detection of DENV and CHIKV. **a** Clinical study design: A total of 236 suspected patients were enrolled, all evaluated according to clinical diagnostic guidelines, including RT-qPCR, DENV NS1 antigen testing, and anti-CHIKV IgM detection. **b**, **c** Box plots showing the distribution of fluorescence signals (RFU) and lateral flow strip intensities (Dr) for DENV (**b**) and CHIKV (**c**) positive samples compared to negative controls (*n* = 50). Red dashed lines indicate the cut-off values. **d**, **e** Consistency analysis of the duplex RT-MIRA assay compared to standard methods for DENV (**d**) and CHIKV (**e**) diagnosis. Tables summarize sensitivity, specificity, positive/negative predictive values (PPV/NPV), overall agreement (Kappa coefficient) and McNemar’s test *p*-value are provided. RT-MIRA: Reverse transcription multienzyme isothermal amplification; CHIKV: Chikungunya virus; DENV: Dengue virus; RT-qPCR: Reverse transcription-quantitative polymerase chain reaction

A total of 236 serum samples from patients suspected of DENV and CHIKV infection were subjected to testing using anti-CHIKV IgM, DENV NS1 antigen, and RT-qPCR. The testing process yielded the identification of 78 samples positive for DENV and 18 samples positive for CHIKV, including one co-infection case (Fig. 5a). Subsequently, these identified positive samples, along with 50 negative controls, were analyzed by the duplex RT-MIRA assay. The assay successfully distinguished clinical positives from negatives (Fig. 5b, c), demonstrating a sensitivity of 96.15% (75/78; 95% *CI*: 89.28%–98.67%) and specificity of 100% (50/50; 95% *CI*: 92.87%–100%) for DENV. For CHIKV, the sensitivity and specificity were 88.89% (16/18; 95% *CI*: 67.20%–96.90%) and 100% (95% *CI*: 92.87%–100%), respectively (Fig. 5b–e). Statistical analysis revealed substantial agreement between Duplex RT-MIRA and RT-qPCR for DENV detection (*κ* = 0.96; *P* > 0.05), as well as strong concordance with the DENV NS1 antigen (*κ* = 0.83; *P* > 0.05) (Fig. 5d). For CHIKV, while agreement with RT-qPCR was excellent (*κ* = 0.92; *P* > 0.05), concordance with anti-CHIKV IgM was moderate (*κ* = 0.53; *P* > 0.05) (Fig. 5e). Further analysis of cycle threshold (CT) values confirmed duplex RT-MIRA robust detection across the tested viral loads, with only a single discordant case for DENV (Fig. S4a). Additionally, discrepancies between molecular and serological results were largely attributed to sampling timing, reflecting the distinct diagnostic windows of the two methods (Fig. S4b).

**Fig. 6:**
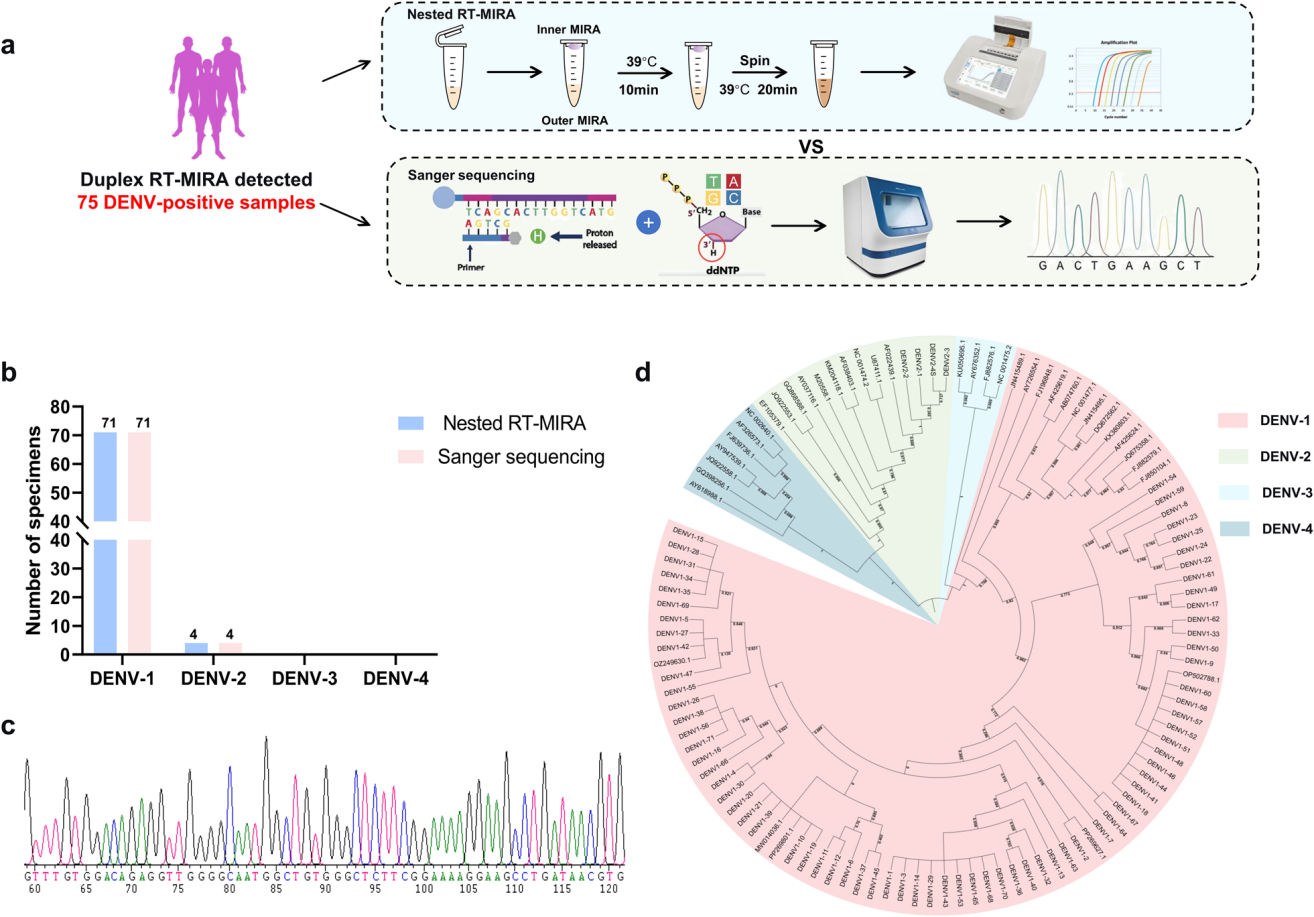
Clinical evaluation of the serotyping accuracy of nested RT-MIRA for DENV. **a** Seventy-five DENV-positive samples were tested using nested RT-MIRA, with Sanger sequencing performed in parallel. **b** Comparison of DENV serotyping results obtained by nested RT-MIRA and Sanger sequencing **c** Representative sequencing chromatogram of the DENV E region. **d** Nucleotide sequences of the amplified DENV E region were aligned using MAFFT v.7, and a phylogenetic tree was constructed with IQ-TREE v.2.2.2.6 using the GTR + F + I + R4 model, with 1,000 ultrafast bootstrap replicates. RT-MIRA: Reverse transcription multienzyme isothermal amplification; DENV: Dengue virus

Subsequently, nested RT-MIRA was performed on 75 DENV-positive samples for serotyping and Sanger sequencing (Fig. 6a). The results indicated that 71 samples were positive for DENV-1 (96.7%), while 4 samples were positive for DENV-2 (5.3%) (Fig. 6b). The validity of these findings was further substantiated by Sanger sequencing results and a phylogenetic tree based on the E1 region, which provided additional validation of the accuracy of the findings (Fig. 6c, d).

### External validation

The external validation results demonstrated that the duplex RT-MIRA assay effectively detected all 10 cases of CHIKV mono-infection and 2 cases of CHIKV/DENV co-infection (Fig. S5). In the analysis of artificially simulated samples, the assay accurately identified specific DENV serotypes and successfully distinguished between the four DENV serotypes even in the presence of CHIKV co-infection within complex matrices (Fig. S6). Overall, these findings confirmed the high reliability and clinical applicability of the duplex RT-MIRA assay for the accurate detection of CHIKV and DENV, as well as the high accuracy of the nested RT-MIRA assay for DENV serotyping.

## Discussion

The present study introduced a duplex POCT platform designed for the simultaneous detection and differentiation of the DENV and the CHIKV, along with a nested RT-MIRA method for DENV serotyping. The duplex RT-MIRA method demonstrated ultra-sensitive detection capabilities, achieving LOD95 of 13.47 copies/μl for DENV and 10.49 copies/μl for CHIKV. This performance surpassed that of existing methods such as RPA-CRISPR/Cas12a and RT-LAMP [16–18].

In order to enhance the accuracy of the detection process, the study investigated the cross-reactivity of the test with 35 similar pathogens. The nested RT-MIRA assay exhibited LOD95 of 9.17, 9.13, 2.03, and 1.6 copies/μl for DENV serotypes 1–4, thus demonstrating superior sensitivity in comparison to various RT-LAMP assays [19, 20]. Clinical validation demonstrated that the duplex RT-MIRA assay exhibited a sensitivity of 96.15% and a specificity of 100% for the detection of DENV, thereby surpassing the RT-RPA method that was enhanced by magnetic field agglutination [21]. Notably, this high accuracy extended to serotyping: all 75 DENV-positive samples were correctly typed, with results fully consistent with Sanger sequencing. For CHIKV, the sensitivity and specificity levels were recorded as 88.89% and 100%, respectively. However, it is important to note that only 18 CHIKV-positive clinical samples were included in the study, which may explain the slightly lower sensitivity observed for CHIKV. Our temporal analysis of discordant cases (Fig. S4b) confirms that discrepancies between molecular and serological results stem from their distinct diagnostic windows rather than assay failure. Duplex RT-MIRA-positive/Serology-negative samples were clustered in the early acute phase (median 1–2 days), reflecting the viremic window before seroconversion. In contrast, duplex RT-MIRA-negative/Serology-positive samples occurred later (median 5–6 days), consistent with viral clearance and the subsequent immune response. These findings underscore the utility of duplex RT-MIRA for early diagnosis, while serology serves as a complementary tool for later stages, aligning with established infection kinetics [22, 23].

As summarized in Table S6, the RT-MIRA platform demonstrates distinct advantages over conventional qPCR and other isothermal methods (e.g., RT-LAMP) in terms of operational simplicity, speed, and cost-effectiveness. With a rapid detection time of 20–30 min and a moderate cost ($5–10 per test), it offers a scalable solution for resource-limited settings without the need for sophisticated thermocyclers [24–29, 35–39]. The entire workflow, encompassing automated sample preparation and result interpretation, can be completed within 40 min. However, when compared to the integrated “extraction-amplification-detection” design concept emphasized by molecular POCT platforms, particularly those with extraction-free workflows, further optimization is necessary [30, 31] . Despite the success of accelerated virus lysis techniques, such as heating specimens at 80 °C for 2 min, in facilitating nucleic acid extraction from non-invasive materials such as saliva and urine, the isolation and detection of viral RNA from blood samples remains a challenging endeavor [32]. This phenomenon can be attributed to the presence of immunoglobulin G and various enzymatic inhibitors, including anticoagulants, hemoglobin, and RNases, which have the capacity to degrade RNA [33, 34]. To address this issue, the study employed fully automated equipment to facilitate viral RNA release and purification, thereby achieving enhanced detection sensitivity.

This study has several limitations. First, although we conducted preliminary external validation and simulated co-infection testing to assess the assay's feasibility, the number of clinical samples was relatively small. This was especially true for naturally occurring co-infections of the DENV and the CHIKV, as well as specific DENV serotypes (DENV-2, −3, and −4). Consequently, this method was validated in only one laboratory, so inter-laboratory or multicenter validation was not possible. Second, we evaluated the assay using serum and plasma but did not assess the feasibility of using whole blood or finger-prick samples, which are critical for field applications. Third, while the current workflow is rapid, it is not yet fully automated. Future work will focus on conducting large-scale, multicenter validations with diverse cohorts and developing a fully integrated "sample-to-answer" device that combines sample pretreatment, amplification, and smart terminal connectivity.

## Conclusions

In summary, we have developed a rapid, duplex RT-MIRA diagnostic technique that can detect both DENV and CHIKV simultaneously. For samples testing positive for DENV, the nested RT-MIRA method enables accurate serotyping. Our results demonstrate that this technique performs consistently well compared to RT-qPCR, providing faster, more convenient results. These qualities make it a suitable candidate for resource-limited settings and a promising tool for future POCT applications.

## Supplementary Information

Additional file 1.

Additional file 2.

Additional file 3.

Additional file 4.

Additional file 5.

Additional file 6.

Additional file 7.

Additional file 8.

Additional file 9.

Additional file 10.

Additional file 11.

Additional file 12.

## Data Availability

Data will be made available on request.

## Abbreviations

BHK-21: Baby hamster kidney-21
BHQ1-dT: Internal black hole quencher 1-labeled thymidine
BHQ3-dT: Internal black hole quencher 3-labeled thymidine
Biotin: Biotinylation
C3 Spacer: 3′-Carbon spacer
CHIKV: Chikungunya virus
*CIs*: Confidence intervals
CRISPR: Clustered regularly interspaced short palindromic repeats
*CV*: Coefficient of variation
CT: Cycle threshold
DENV: Dengue virus
Dig: Digoxigenin
ELISA: Enzyme-linked immunosorbent assay
FAM-dT: Internal carboxyfluorescein-labeled thymidine
idSp: Internal abasic dSpacer (tetrahydrofuran residue)
IgG: Immunoglobulin G
IgM: Immunoglobulin M
*k*: Cohen’s kappa
LFD: Lateral flow dipsticks
LOD: Limit of detection
LOD95: 95% Limit of detection
NAATs: Nucleic acid amplification tests
NS1: Non-structural protein 1
POCT: Point-of-care testing
ROX-dT: Internal carboxy-X-rhodamine-labeled thymidine
RPA: Recombinase polymerase amplification
RT-LAMP: Reverse transcription loop-mediated isothermal amplification
RT-MIRA: Reverse transcription multienzyme isothermal amplification
RT-qPCR: Reverse transcription-quantitative polymerase chain reaction
*SD*: Standard deviation
