# Supplementary figures and images for: A rapid multiplex platform for simultaneous detection of chikungunya virus, dengue virus, and dengue serotyping based on isothermal amplification and lateral flow dipsticks
Source: Infect Dis Poverty. 2026 May 9;15:52. doi: 10.1186/s40249-026-01450-9 (PMC13156856; doi:10.1186/s40249-026-01450-9)

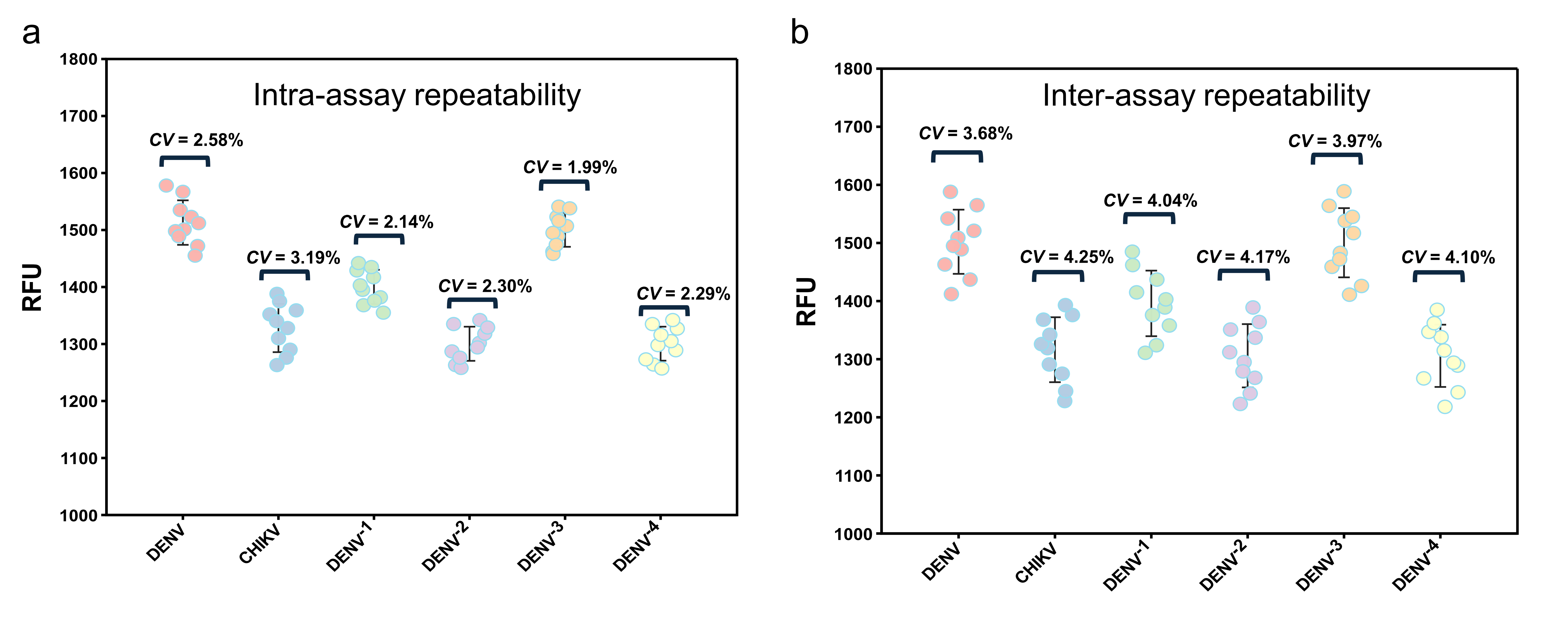

Supplement: Supplementary file 3 — Additional file 3. [file 40249_2026_1450_MOESM3_ESM.tif]

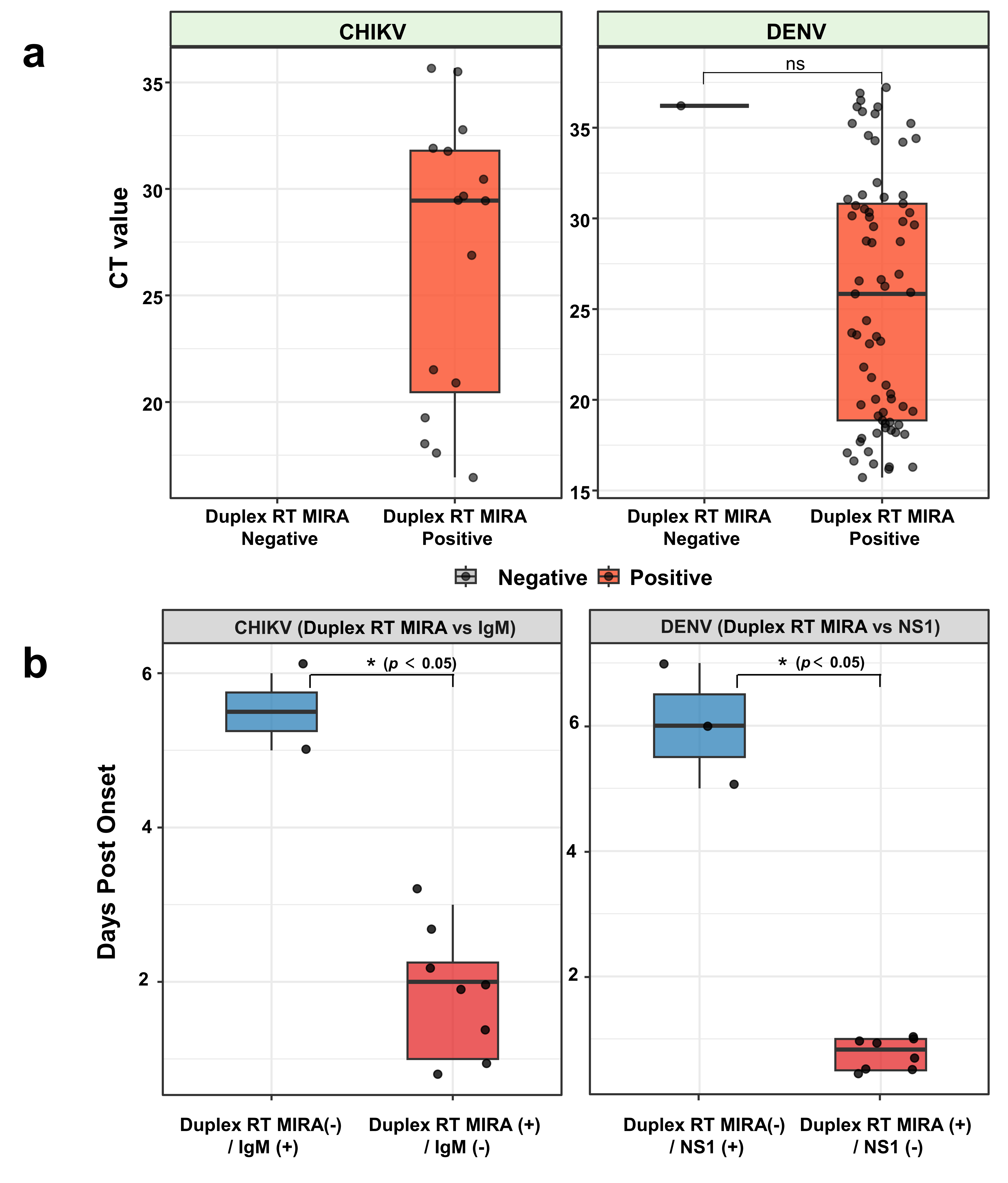

Supplement: Supplementary file 4 — Additional file 4. [file 40249_2026_1450_MOESM4_ESM.tif]

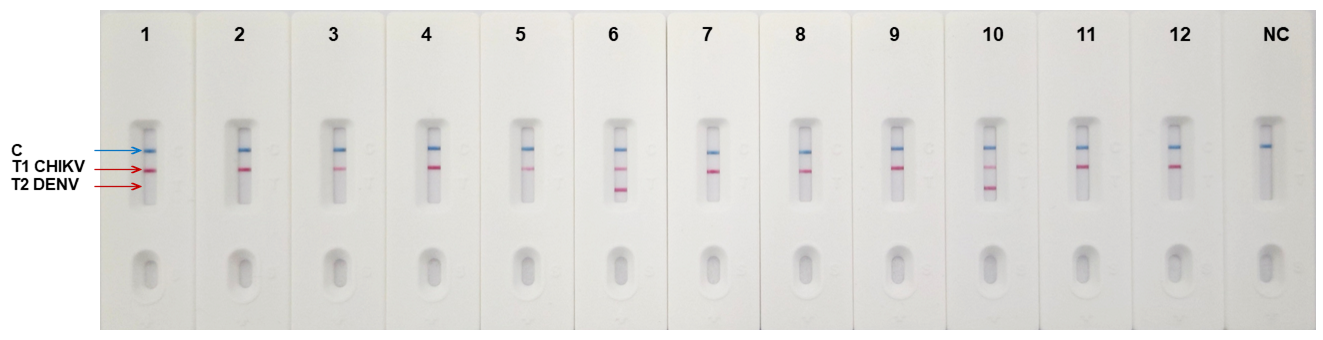

Supplement: Supplementary file 5 — Additional file 5. [file 40249_2026_1450_MOESM5_ESM.tif]
