# Supplementary material for: A rapid multiplex platform for simultaneous detection of chikungunya virus, dengue virus, and dengue serotyping based on isothermal amplification and lateral flow dipsticks
Source: Infect Dis Poverty. 2026 May 9;15:52. doi: 10.1186/s40249-026-01450-9 (PMC13156856; doi:10.1186/s40249-026-01450-9)
Supplement: Supplementary file 6 — Additional file 6. [file 40249_2026_1450_MOESM6_ESM.docx]

**Table S1** Basic information and test results of the pathogens used for specific evaluation in this study

| **Species** | **Type** | **Strain/clinical sample** | **Duplex RT-MIRA assay** | **Nested RT-MIRA serotyping** |
| --- | --- | --- | --- | --- |
|  |  |  | **Result** | **Result** |
| Chikungunya virus | RNA virus | Ross strain (ECSA) | + | - |
|  |  | 181/25 strain (Asia) | + | - |
|  |  | LR2006_OPY1 strain (IOL) | + | - |
|  |  | 37997 strain (WA) | + | - |
| Zika virus |  | GZ-01 | - | - |
| Dengue virus serotype 1 |  | Clinically isolated strain | + | Only DENV-1（+） |
| Dengue virus serotype 2 |  | Clinically isolated strain | + | Only DENV-2（+） |
| Dengue virus serotype 3 |  | Clinically isolated strain | + | Only DENV-3（+） |
| Dengue virus serotype 4 |  | Clinically isolated strain | + | Only DENV-4（+） |
| Japanese encephalitis virus |  | ATCC SA14 - 2 | - | - |
| Ross River virus |  | T48/DQ226993 | - | - |
| O'nyong-nyong virus |  | An D SS 234 | - | - |
| West Nile virus |  | VR-3198SD | - | - |
| influenza A virus |  | Clinical sample | - | - |
| influenza B virus |  | Clinical sample | - | - |
| Human immunodeficiency virus type 1 |  | Clinical sample | - | - |
| Hepatitis C virus |  | Clinical sample | - | - |
| Yellow fever virus |  | Clinical sample | - | - |
| Measles virus |  | Clinical sample | - | - |
| Rubella virus |  | Clinical sample | - | - |
| Hepatitis B virus | DNA virus | Clinical sample | - | - |
| Cytomegalovirus |  | Clinical sample | - | - |
| Mumps virus |  | Clinical sample | - | - |
| Epstein - Barr virus |  | Clinical sample | - | - |
| BK virus |  | Clinical sample | - | - |
| Staphylococcus aureus | Bacteria | Clinical sample | - | - |
| Pseudomonas aeruginosa |  | Clinical sample | - | - |
| Escherichia coli |  | Clinical sample | - | - |
| Klebsiella pneumoniae |  | Clinical sample | - | - |
| Acinetobacter baumannii |  | Clinical sample | - | - |
| Streptococcus pyogenes |  | Clinical sample | - | - |
| Listeria monocytogenes |  | Clinical sample | - | - |
| Trichosporon asahii | Fungi | Clinical sample | - | - |
| Pneumocystis jirovecii |  | Clinical sample | - | - |
| Trichophyton mentagrophytes |  | Clinical sample | - | - |
| Candida albicans | Yeasts | Clinical sample | - | - |
| Cryptococcus neoformans |  | Clinical sample | - | - |
| Candida parapsilosis |  | Clinical sample | - | - |
