# Supplementary material for: A rapid multiplex platform for simultaneous detection of chikungunya virus, dengue virus, and dengue serotyping based on isothermal amplification and lateral flow dipsticks
Source: Infect Dis Poverty. 2026 May 9;15:52. doi: 10.1186/s40249-026-01450-9 (PMC13156856; doi:10.1186/s40249-026-01450-9)
Supplement: Supplementary file 7 — Additional file 7. [file 40249_2026_1450_MOESM7_ESM.docx]

**Table S2** DENV and CHIKV duplex RT-MIRA primer probe sequences used in this study

| **Primer name** | **Sequence 5′–3′** | **Target gene** | **Position*** | **Length**  **(bp)** |
| --- | --- | --- | --- | --- |
| DENV-F1# | GCATATTGACGCTGGGAGAGACCAGAGATCC | 3’-UTR | 10632-10662 | 31 |
| DENV-F2 | AACAGCATATTGACGCTGGGAGAGACCAGA |  | 10628-10657 | 30 |
| DENV-F3 | TGCAAAGGACTAGAGGTTAGAGGAGACCCC |  | 10585-10614 | 30 |
| DENV-R1# | CCATTTTSTGGCGTTCTGTGCCTGGAATGA |  | 10679-10708 | 30 |
| DENV-R2 | GAACCTGTTGATTCAACAGCACCATTCCA |  | 10706-10734 | 29 |
| DENV-F1-Biotin# | Biotin-GCATATTGACGCTGGGAGAGACCAGAGATCC |  | 10632-10662 | 31 |
| DENV-P-DIG-nfo# | Dig-TTSTGGCGTTCTGTGCCTGGAATGATGCT/idSp/TAGAG  ACAGCAGGAT-C3 Spacer |  | 10659-10703 | 45 |
| DENV-P-exo# | TTSTGGCGTTCTGTGCCTGGAATGA/ROX-dT/GCT/idSp//BHQ3-dT/AGAGACAGCAGGAT- C3 Spacer |  | 10659-10703 | 45 |
| CHIKV-F1 | GAACGCCGTCCTCCTRCCCAAYGTRCATA | NSP4 | 6667-6695 | 29 |
| CHIKV-F2 | GATTTCGATGCCATYATAGCCGCACRCTTTYA |  | 6719-6749 | 32 |
| CHIKV-F3# | CACTATTTGAYATGTCTGCCGAGGAYTTCGATGC |  | 6696-6729 | 34 |
| CHIKV-F4 | CGAGGAYTTCGATGCCATYATAGCCGCACAC |  | 6715-6745 | 31 |
| CHIKV-R1# | AAGCGCGTACCTGTCGGYAGRTGACAGCTG |  | 6901-6930 | 30 |
| CHIKV-R2 | GTGTTGACGAACAGAGTTAGGAACATACC |  | 6956-6984 | 29 |
| CHIKV-R2 | Biotin-GTGTTGACGAACAGAGTTAGGAACATACC |  | 6956-6984 | 29 |
| CHIKV-P-nfo# | FAM-CGGACATAGCCTCCTTTGATAAGAGCCAA/idSp/TGAT  TCACTTGCGCTTAC-C3 Spacer |  | 6774-6822 | 48 |
| CHIKV-P-exo# | CGGACATAGCCTCCTTTGATAAGAGCCAA/FAM-dT/T/idSp/A/BHQ1-dT/TCACTTGCGCTTAC-C3 Spacer |  | 6774-6822 | 48 |

*NCBI Reference Sequence: DENV: (EF025110.1); CHIKV: (NC_004162.2.); # Indicates the information of the best primer/probe sequence that has been finally selected. FAM-dT: internal Carboxyfluorescein-labeled thymidine; ROX-dT: internal Carboxy-X-rhodamine-labeled thymidine; BHQ1-dT: internal Black Hole Quencher 1-labeled thymidine; BHQ3-dT: internal Black Hole Quencher 3-labeled thymidine; idSp: internal abasic dSpacer (tetrahydrofuran residue); Dig: digoxigenin; Biotin: biotinylation; C3 Spacer: 3'-carbon spacer.
