# Supplementary material for: A rapid multiplex platform for simultaneous detection of chikungunya virus, dengue virus, and dengue serotyping based on isothermal amplification and lateral flow dipsticks
Source: Infect Dis Poverty. 2026 May 9;15:52. doi: 10.1186/s40249-026-01450-9 (PMC13156856; doi:10.1186/s40249-026-01450-9)
Supplement: Supplementary file 8 — Additional file 8. [file 40249_2026_1450_MOESM8_ESM.docx]

**Table S3** DENV serotyping nested RT-MIRA primer and probe sequences used in this study

| **Primer name** | **Sequence5′–3′** | **Target gene** | **Position*** | **Length**  **(bp)** |
| --- | --- | --- | --- | --- |
| DENV1-F1# | GTGGGGATGTAAAAACCYGGGAGGCTGCAA | 3’-UTR | 10449-10478 | 30 |
| DENV1-F2 | ACCGTGCTGCCTGTRGCTCCATCGTGGGGA |  | 10426-10455 | 30 |
| DENV1-F3 | TCAGGCCGAAAGCCACGGYTTGAGCAAACC |  | 10399-10428 | 30 |
| DENV1-R1 | TCTAACCTCTAGTCCTTACCACCAGGGTAC |  | 10576-10605 | 30 |
| DENV1-R2 | GGGTCTCCTCTAACCTCTAGTCCTTACCAC |  | 10584-10613 | 30 |
| DENV1-R3# | CAGCGTCAATATGCTGTTTRTTGTTRTGCG |  | 10616-10645 | 30 |
| DENV1-WF1 | AGGACGTAAAATGAAGTCAGGCCGAAAGCC |  | 10383-10412 | 30 |
| DENV1-WF2# | AAGAGCTATGCTGCCTGTGAGCCCCGTCYA |  | 10353-10382 | 30 |
| DENV1-WF3 | ATAGTACGGTAARAGCTATGCTGCCTGTGA |  | 10343-10372 | 30 |
| DENV1-WR1 | AGCGTCAATATGCTGTTTATTGTTRTGCGG |  | 10615-10644 | 30 |
| DENV1-WR2# | CAGGATCTCTGGTCTCTCCCAGCGTCAATA |  | 10635-10664 | 30 |
| DENV1-WR3 | GATGCTGTAGAGACAGCAGGATCTCTGGTC |  | 10651-10680 | 30 |
| DENV1-R3-Biotin# | Biotin-CAGCGTCAATATGCTGTTTRTTGTTRTGCG |  | 10616-10645 | 30 |
| DENV1-WR2-Biotin | Biotin-CAGGATCTCTGGTCTCTCCCAGCGTCAATA |  | 10635-10664 | 30 |
| DENV1-P-FAM-nfo# | FAM-AGGCTGCAACCCATGGAAGCTGTACGCATGG/idSp/GTAGCA  GACTAGTGG-C3 Spacer |  | 10470-10516 | 47 |
| DENV1-P-exo# | AGGCTGCAACCCATGGAAGCTGTACGCA/FAM-dT/GG/idSp/G  /BHQ1-dT/AGCAGACTAGTGG-C3 Spacer |  | 10470-10516 | 47 |
| DENV2-F1 | ACGCCTTTCAATATGCTGAAACGCGAGAGA | 5’-UTR - C | 127-156 | 30 |
| DENV2-F2 | AGAGAGCAGATCTCTGATGAATAACCAACG |  | 81-110 | 30 |
| DENV2-F3# | CCTTTCAATATGCTGAAACGCGAGAGAAAC |  | 130-159 | 30 |
| DENV2-R1# | GGCCATGAACAGTTTYARYGKTCCKCGTCC |  | 214-243 | 30 |
| DENV2-R2 | AAACGAAGGAAHGCCACHARGGCCATGAA |  | 235-263 | 29 |
| DENV2-R3 | CCTGCTGTTGGYGGGATTGTTAGGAAACG |  | 259-287 | 29 |
| DENV2-WF1 | TTAGAGAGCAGATCTCTGATGAATAACCAA |  | 79-108 | 30 |
| DENV2-WF2# | TTTAWTTAGAGAGCAGATCTCTRATGAATA |  | 74-103 | 30 |
| DENV2-WF3# | AGGRAGCTAAGCTYAACGTAGTTCTAACAGT |  | 41-71 | 31 |
| DENV2-WR1 | CGAAGGAAHGCCACHARGGCCATGAACAGTTT |  | 229-260 | 32 |
| DENV2-WR2 | GGATTGTYAGGAAACGAAGGAAHGCCACYA |  | 245-274 | 30 |
| DENV2-WR3 | TGTTGGYGGGATTGTTAGGAAACGAAGGAA |  | 253-282 | 30 |
| DENV2-R1-Biotin# | Biotin-GGCCATGAACAGTTTYARYGKTCCKCGTCC |  | 214-243 | 30 |
| DENV2-WR3-Biotin | Biotin-TGTTGGYGGGATTGTTAGGAAACGAAGGAA |  | 253-282 | 30 |
| DENV2-P-FAM-nfo# | FAM-CTGAAACGCGAGAGAAACCGCGTGTCRACT/idSp/TGCARCA  GCTGACRAA-C3 Spacer |  | 142-188 | 47 |
| DENV2-P-exo# | CTGAAACGCGAGAGAAACCGCGTGTCRAC/FAM-dT//idSp//BHQ1-dT/GCARCAGCTGACRAA-C3 Spacer |  | 142-188 | 47 |
| DENV3-F1# | GAGAGCAGATYTCTGATGAACAACCAACGG | 5’-UTR - C | 80-109 | 30 |
| DENV3-F2 | TTTTTATTAGAGAGCAGATYTCTGATGAAC |  | 71-100 | 30 |
| DENV3-F3 | CAGTTTCGACTCGGAAGCTTGCTTAACGTA |  | 30-59 | 30 |
| DENV3-R1 | TTCAGCARTCCYYTTGAGAATCTCTTCGCC |  | 181-210 | 30 |
| DENV3-R2# | GCTATGAACGCCATRACCAATTTCATTRGTCC |  | 218-249 | 32 |
| DENV3-R3 | TGGCYAGAAATCTRAGGAARGCTATGAACGC |  | 239-269 | 31 |
| DENV3-WF1# | CTCGGAAGCTTGCTTAACGTAGTGCTGACAG |  | 39-69 | 31 |
| DENV3-WF2 | CGTGGACCGACAAGAACAGTTTCGACTCGG |  | 14-43 | 30 |
| DENV3-WF3 | TTGTTAGTCTACGTGGACCGACAAGAACAG |  | 3-32 | 30 |
| DENV3-WR1 | GCCAARAYTCCYGCTGTYGGTGGRATGGC |  | 266-294 | 29 |
| DENV3-WR2 | CCYGCTGTYGGTGGRATGGCYAGAAATCT |  | 257-285 | 29 |
| DENV3-WR3# | GAAGGTTCCCCATCTAGCCAAGACTCCTGC |  | 281-310 | 30 |
| DENV3-R2-Biotin# | Biotin-GCTATGAACGCCATRACCAATTTCATTRGTCC |  | 218-249 | 32 |
| DENV3-WR3-Biotin | Biotin- GAAGGTTCCCCATCTAGCCAAGACTCCTGC |  | 281-310 | 30 |
| DENV3-P-FAM-nfo# | FAM-AACGCGTGAGAAACCGTGTGTCAACTGGATCAC/idSp/GTT  GGCGAAGAGAT-C3 Spacer |  | 144-191 | 48 |
| DENV3-P-exo# | AACGCGTGAGAAACCGTGTGTCAACTGGA/FAM-dT/CAC/idSp/G  /BHQ1-dT/TGGCGAAGAGAT-C3 Spacer |  | 144-191 | 48 |
| DENV4-F1 | CGAGARAAGCGCTCAGTAGCTYTAACACCA | prM-E | 700-729 | 30 |
| DENV4-F2 | ACCGAACCYGAAGACATTGAYTGCTGGTG |  | 613-641 | 29 |
| DENV4-F3# | AAGACATTGAYTGCTGGTGCAAYCTCACGTC |  | 623-653 | 31 |
| DENV4-R1# | GCRAATCCTGGGTTTCTGAGTATCCARCTYTC |  | 808-839 | 32 |
| DENV4-R2 | GTYCGCTGGATYCCTGTTTGCCCRATCAT |  | 865-893 | 29 |
| DENV4-R3 | GTTTCTGAGTATCCARCTYTCYACYCTCTG |  | 799-828 | 30 |
| DENV4-WF1# | AAYACCGAACCYGAAGACATTGAYTGCTGG |  | 610-639 | 30 |
| DENV4-WF2 | ATGGAYYTGGGTGARATGTGYGARGACAC |  | 553-581 | 29 |
| DENV4-WF3 | GGGAGACCTCTCTTGTTYAARACAACAGA |  | 496-524 | 29 |
| DENV4-WR1 | GCRAATCCTGGGTTTCTGAGTATCCARCTYTC |  | 808-839 | 32 |
| DENV4-WR2# | GATYCCTGTTTGCCCRATCATRTARGCCAT |  | 856-885 | 30 |
| DENV4-WR3 | TYCGCTGGATYCCTGTTTGCCCRATCATRTA |  | 862-892 | 31 |
| DENV4-R1-Biotin# | Biotin-GCRAATCCTGGGTTTCTGAGTATCCARCTYTC |  | 808-839 | 32 |
| DENV4-WR2-Biotin | Biotin-GATYCCTGTTTGCCCRATCATRTARGCCAT |  | 856-885 | 30 |
| DENV4-P-FAM-nfo# | FAM-TGGGATTGGARACAAGRGCTGARACATGGAT/idSp/TCATCG  GAAGGRGCT-C3 Spacer |  | 740-786 | 47 |
| DENV4-P-exo# | TGGGATTGGARACAAGRGCTGARACATGGA/FAM-dT//idSp//BHQ1-dT/CATCGGAAGGRGCT-C3 Spacer |  | 740-786 | 47 |

*NCBI Reference Sequence: DENV 1: (KC692517.1); DENV 2 (KR920365.1); DENV 3 (KJ737430.1); DENV 4 (KY451945.1); # Indicates the information of the best primer/probe sequence that has been finally selected. FAM-dT: internal Carboxyfluorescein-labeled thymidine; BHQ1-dT: internal Black Hole Quencher 1-labeled thymidine; idSp: internal abasic dSpacer (tetrahydrofuran residue); DIG: digoxigenin; Biotin: biotinylation; C3 Spacer: 3'-carbon spacer.
