# Supplementary material for: A rapid multiplex platform for simultaneous detection of chikungunya virus, dengue virus, and dengue serotyping based on isothermal amplification and lateral flow dipsticks
Source: Infect Dis Poverty. 2026 May 9;15:52. doi: 10.1186/s40249-026-01450-9 (PMC13156856; doi:10.1186/s40249-026-01450-9)
Supplement: Supplementary file 9 — Additional file 9. [file 40249_2026_1450_MOESM9_ESM.docx]

**Table S4** The results of reagent stability assessment

| **Sample ID** | **Storage**  **Temperature (°C)** | **Storage time (days)** | | | | | | | |
| --- | --- | --- | --- | --- | --- | --- | --- | --- | --- |
|  |  | **0** | | | **7** | | | **14** | |
| Sample #1  (1 × 10^5^ copies/μl) | 4 | DENV (+) | CHIKV (+) | DENV (+) | | CHIKV (+) | DENV (+) | | CHIKV (+) |
|  |  | DENV (+) | CHIKV (+) | DENV (+) | | CHIKV (+) | DENV (+) | | CHIKV (+) |
|  |  | DENV (+) | CHIKV (+) | DENV (+) | | CHIKV (+) | DENV (+) | | CHIKV (+) |
| Sample #2  (1 × 10^2^ copies/μl) | 4 | DENV (+) | CHIKV (+) | DENV (+) | | CHIKV (+) | DENV (+) | | CHIKV (+) |
|  |  | DENV (+) | CHIKV (+) | DENV (+) | | CHIKV (+) | DENV (+) | | CHIKV (+) |
|  |  | DENV (+) | CHIKV (+) | DENV (+) | | CHIKV (+) | DENV (+) | | CHIKV (+) |
| Sample #1  (1 × 10^5^ copies/μl) | 25 | DENV (+) | CHIKV (+) | DENV (+) | | CHIKV (+) | DENV (+) | | CHIKV (+) |
|  |  | DENV (+) | CHIKV (+) | DENV (+) | | CHIKV (+) | DENV (+) | | CHIKV (+) |
|  |  | DENV (+) | CHIKV (+) | DENV (+) | | CHIKV (+) | DENV (+) | | CHIKV (+) |
| Sample #2  (1 × 10^2^ copies/μl) | 25 | DENV (+) | CHIKV (+) | DENV (+) | | CHIKV (+) | DENV (+) | | CHIKV (+) |
|  |  | DENV (+) | CHIKV (+) | DENV (+) | | CHIKV (+) | DENV (+) | | CHIKV (+) |
|  |  | DENV (+) | CHIKV (+) | DENV (+) | | CHIKV (+) | DENV (+) | | CHIKV (+) |
| Sample #1  (1 × 10^5^ copies/μl) | 37 | DENV (+) | CHIKV (+) | DENV (+) | | CHIKV (+) | DENV (+) | | CHIKV (+) |
|  |  | DENV (+) | CHIKV (+) | DENV (+) | | CHIKV (+) | DENV (+) | | CHIKV (+) |
|  |  | DENV (+) | CHIKV (+) | DENV (+) | | CHIKV (+) | DENV (+) | | CHIKV (+) |
| Sample #2  (1 × 10^2^ copies/μl) | 37 | DENV (+) | CHIKV (+) | DENV (+) | | CHIKV (+) | DENV (+) | | CHIKV (+) |
|  |  | DENV (+) | CHIKV (+) | DENV (+) | | CHIKV (+) | DENV (+) | | CHIKV (+) |
|  |  | DENV (+) | CHIKV (+) | DENV (+) | | CHIKV (+) | DENV (+) | | CHIKV (+) |
