# Supplementary material for: A rapid multiplex platform for simultaneous detection of chikungunya virus, dengue virus, and dengue serotyping based on isothermal amplification and lateral flow dipsticks
Source: Infect Dis Poverty. 2026 May 9;15:52. doi: 10.1186/s40249-026-01450-9 (PMC13156856; doi:10.1186/s40249-026-01450-9)
Supplement: Supplementary file 10 — Additional file 10. [file 40249_2026_1450_MOESM10_ESM.docx]

**Table S5** The results of duplex RT-MIRA anti-interference ability studies

| **Interfering substances** | **Concentration of interfering substances (results)** | | | | | |
| --- | --- | --- | --- | --- | --- | --- |
|  | **Low** | **results** | **Medium** | **results** | **High** | **results** |
| Antiviral drug: Ribavirin | 25 μg/ml | DENV (+) CHIKV (+) | 50 μg/ml | DENV (+) CHIKV (+) | 100 μg/ml | DENV (+) CHIKV (+) |
| Acetaminophen | 1 mg/l | DENV (+) CHIKV (+) | 5 mg/l | DENV (+) CHIKV (+) | 10 mg/l | DENV (+) CHIKV (+) |
| Antibiotic: amoxicillin | 1 mg/l | DENV (+) CHIKV (+) | 5 mg/l | DENV (+) CHIKV (+) | 10 mg/l | DENV (+) CHIKV (+) |
| Hormonal drugs: dexamethasone | 5 mg/l | DENV (+) CHIKV (+) | 10 mg/l | DENV (+) CHIKV (+) | 20 mg/l | DENV (+) CHIKV (+) |
| Heparin | 5 mg/ml | DENV (+) CHIKV (+) | 15 mg/ml | DENV (+) CHIKV (+) | 30 mg/ml | DENV (+) CHIKV (+) |
| EDTA | 1 μg/ml | DENV (+) CHIKV (+) | 5 μg/mLl | DENV (+) CHIKV (+) | 10 μg/ml | DENV (+) CHIKV (+) |
| Sodium citrate | 0.1 mg/ml | DENV (+) CHIKV (+) | 0.3 mg/ml | DENV (+) CHIKV (+) | 0.6 mg/ml | DENV (+) CHIKV (+) |
| Hemoglobin | 5 mg/ml | DENV (+) CHIKV (+) | 10 mg/ml | DENV (+) CHIKV (+) | 20 mg/ml | DENV (+) CHIKV (+) |
| Albumin | 5 μg/ml | DENV (+) CHIKV (+) | 10 μg/ml | DENV (+) CHIKV (+) | 20 μg/ml | DENV (+) CHIKV (+) |
