# Supplementary material for: A rapid multiplex platform for simultaneous detection of chikungunya virus, dengue virus, and dengue serotyping based on isothermal amplification and lateral flow dipsticks
Source: Infect Dis Poverty. 2026 May 9;15:52. doi: 10.1186/s40249-026-01450-9 (PMC13156856; doi:10.1186/s40249-026-01450-9)
Supplement: Supplementary file 11 — Additional file 11. [file 40249_2026_1450_MOESM11_ESM.docx]

**Table S6** Comparison of our method with several chikungunya virus and dengue virus detection methods

| **NO.** | **Pathogens** | **Amplification methods** | **Target** | **Sample type** | **Number of clinical samples** | **Lowest limit of detection** | **Sensitivity**  **(%)** | **Specificity**  **(%)** | **Detection time (min)*** | **Cost of testing ($)** | **Equipment** | **Reference** |
| --- | --- | --- | --- | --- | --- | --- | --- | --- | --- | --- | --- | --- |
| 1 | CHIKV | RT-qPCR | E1 | Serum | 48 | 3.95 RNA copies/ reaction | 79 | 100 | 90-120 | Medium  (5-10$) | Thermocycler | [24] |
| 2 | CHIKV | RT-qPCR | NSP1 | Serum | 47 | 2.1 RNA copies/μl | 100 | 100 | 90-120 | Medium  (5-10$) | Thermocycler | [25] |
| 3 | CHIKV | RT-qPCR | NSP1 | Serum | 10 | 0.5 PFU | 100 | 100 | 90-120 | Medium  (5-10$) | Thermocycler | [26] |
| 4 | CHIKV | CRISPR-Cas13a SHERLOCK | NSP1 | plasma | 146 | 215 copies/reaction | 94.52 | 100 | ~60 | High  (>15$) | Fluorometer | [27] |
| 5 | CHIKV | RT-RPA | NSP1 | plasma | 78 | 80 copies/reaction | 100 | 100 | 20 | Medium  (5-10$) | Heat Block | [13] |
| 6 | CHIKV | RT-LAMP | E1 | Serum | 35 | 163 molecules | 100 | 80 | 30-40 | Low  (<$5) | Heat Block | [14] |
| 7 | CHIKV | RT-LAMP | E1 | Serum | 100 | 1.18 PFU | 100 | 96.72 | 35 | Low  (<$5) | Heat Block | [28] |
| 8 | CHIKV | ELISA | E2 | Serum | 225 | n.a | 60 | 96.2 | 120–240 | Medium  (5-10$) | Plate Reader | [29] |
| 9 | DENV | LAMP | 5'-UTR & 3'-UTR | Serum | 158 | 800 copies/μl | 96 | 0.95 | 40–60 | Low  (<$5) | Heat Block | [30] |
| 10 | DENV | RPA & CRISPR Cas12a | 3′-UTR | n.a | n.a | 91.7 copies/test | n.a | 100 | 40–60 | High  (>15$) | Fluorometer | [16] |
| 11 | DENV | RT-RAA | 3′-UTR | Serum | 247 | 10 copies/μl | 0.98 | 100 | 20–30 | Medium  (5-10$) | Heat Block | [31] |
| 12 | DENV | RPA | 3′-UTR | Serum | 203 | 50 copies/μl | 77 | 97.9 | 20–30 | Medium  (5-10$) | Heat Block | [32] |
| 13 | DENV & CHIKV | RT-qPCR | n.a | Serum | 60 | 1.56 copies/µl | 100 | 100 | 90-120 | Medium  (5-10$) | Thermocycler | [33] |
| 14 | DENV | Commercial RDT (Antigen) | NS1 | Serum | n.a | ~10⁵ copies/ml | 50–70 | 98 | 15-20 | Low  (<$2) | n.a | [34] |
| 15 | DENV (1-4) | Duplex RT-MIRA | 3'-UTR | Serum | 236 | 13.47copies/μl | 96.15 | 100 | 20 | Medium  (5-10$) | Heat Block | Our method |
|  | CHIKV |  | NSP4 | Serum |  | 10.49 copies/μl | 88.89 | 100 |  |  |  |  |
| 16 | DENV-1 | Nested RT-MIRA | 3'-UTR | Serum | 75 | 9.17 copies/μl | n.a | n.a | 30 | Medium  (5-10$) | Heat Block | Our method |
|  | DENV-2 |  | 5’-UTR - C | Serum |  | 9.13 copies/μl | n.a | n.a |  |  |  |  |
|  | DENV-3 |  | 5’-UTR - C | Serum |  | 2.03 copies/μl | n.a | n.a |  |  |  |  |
|  | DENV-4 |  | prM-E | Serum |  | 1.6 copies/μl | n.a | n.a |  |  |  |  |

Detection time (min) *: Only indicates the time for on-machine testing, excluding the sample pre-treatment process.
