# Supplementary material for: A rapid multiplex platform for simultaneous detection of chikungunya virus, dengue virus, and dengue serotyping based on isothermal amplification and lateral flow dipsticks
Source: Infect Dis Poverty. 2026 May 9;15:52. doi: 10.1186/s40249-026-01450-9 (PMC13156856; doi:10.1186/s40249-026-01450-9)
Supplement: Supplementary file 12 — Additional file 12. [file 40249_2026_1450_MOESM12_ESM.docx]

**Supplementary Information**

Additional file 1: **Table S1.** Basic information and test results of the pathogens used for specific evaluation in this study.

Additional file 2: **Table S2.** DENV and CHIKV duplex RT-MIRA primer probe sequences used in this study.

Additional file 3: **Table S3.** DENV serotyping nested RT-MIRA primer and probe sequences used in this study.

Additional file 4: **Table S4.** The results of the reagent stability assessment.

Additional file 5: **Table S5.** The results of duplex RT-MIRA anti-interference ability studies.

Additional file 6: **Table S6.** Comparison of our method with several chikungunya virus and dengue virus detection methods.

Additional file 7: **Fig S1.** Demographic and clinical characteristics of the study population.

Additional file 8: **Fig S2**. Screening of nested RT-MIRA primers for serotyping of dengue virus.

Additional file 9: **Fig S3.** Repeatability assessment of duplex RT-MIRA and nested RT-MIRA.

Additional file 10: **Fig S4.** Analysis of CT values distribution and time diagnosis window using duplex RT-MIRA detection.

Additional file 11: **Fig S5.** The results of duplex RT-MIRA testing for 12 retrospective CHIKV-positive samples.

Additional file 12: **Fig S6.** Validation of the accuracy of nested RT-MIRA for detecting different serotypes of dengue virus.
